# Carcinoid Heart Disease: Surgical Timing, Right Ventricular Risk Stratification and Operative Strategy

**DOI:** 10.3390/jcdd13060254

**Published:** 2026-06-08

**Authors:** Hani Ali-Ghosh, Jason Kho, Fotios Leventis, Sanjay Asopa, Geoffrey Tsang, Sunil K. Ohri

**Affiliations:** Department of Cardiac Surgery, University Hospital Southampton NHS Foundation Trust, Southampton SO16 6YD, UK; hani.ali-ghosh@doctors.org.uk (H.A.-G.); jason.kho@uhs.nhs.uk (J.K.); fotios.levnetis@uhs.nhs.uk (F.L.); sanjay.asopa@uhs.nhs.uk (S.A.); geoffrey.tsang@uhs.nhs.uk (G.T.)

**Keywords:** carcinoid heart disease, carcinoid syndrome, tricuspid valve replacement, pulmonary valve replacement, surgical timing, right ventricular function, neuroendocrine tumour

## Abstract

Carcinoid heart disease is a progressive right-sided valvulopathy caused by serotonin and other vasoactive mediators released by metastatic neuroendocrine tumours. As oncological therapies have extended survival, cardiac disease has become a leading determinant of mortality. Operative mortality has decreased to 5–6% in contemporary high-volume centres, and long-term survival appears increasingly determined by tumour biology rather than cardiac disease when surgery is appropriately timed. The principal determinant of operative outcome is preoperative right ventricular function; symptom-based referral alone is insufficient because many patients remain compensated until ventricular dysfunction is advanced. This review synthesises the evidence on surgical timing, operative strategy, prosthesis selection, perioperative endocrine management, and emerging transcatheter options. Tricuspid valve replacement is required in the majority of patients, with concomitant pulmonary valve replacement advocated where concurrent disease is present. Bioprosthetic valves are preferred. Continuous perioperative octreotide infusion has substantially reduced the incidence of carcinoid crisis. Structured multidisciplinary decision-making integrating echocardiographic surveillance, biomarker monitoring, and oncological status assessment is essential.

## 1. Introduction

Carcinoid heart disease (CaHD) is a clinically significant complication of metastatic neuroendocrine tumour (NET) disease, occurring in approximately 50–70% of patients with carcinoid syndrome [1,2]. It results from chronic exposure of right-sided cardiac endocardium to serotonin, tachykinins, and transforming growth factor-β released by hepatic metastases, inducing fibrous plaque deposition and progressive tricuspid and pulmonary valve dysfunction [3,4].

Historically, cardiac surgery in CaHD carried perioperative mortality exceeding 20% [5]. Advances in somatostatin analogue therapy and peptide receptor radionuclide therapy (PRRT) have substantially extended survival, and cardiac disease has consequently emerged as a leading determinant of mortality [6,7]. In parallel, improvements in surgical technique, anaesthetic management, and critical care have reduced operative risk substantially. Despite these advances, many patients continue to be referred late, with advanced right ventricular (RV) dysfunction, at which point operative risk is elevated and postoperative recovery is impaired. No validated, CaHD-specific framework for surgical timing currently exists, and clinical decision-making relies on extrapolation from general valve disease guidelines and expert consensus [8,9].

This narrative review synthesises the published evidence on surgical timing, operative strategy, prosthesis selection, perioperative management, and emerging transcatheter options in CaHD, and proposes structured decision frameworks for contemporary multidisciplinary practice. The literature was identified through PubMed, Embase, and Cochrane Library searches using the terms “carcinoid heart disease,” “neuroendocrine tumour cardiac surgery,” and “carcinoid valve surgery,” supplemented by reference list review. Studies were prioritised on the basis of sample size, recency, and relevance to surgical decision-making. As a narrative review, formal systematic methodology was not employed, and selection reflects the authors’ judgement. Throughout this review, recommendations are distinguished as based on published evidence, expert consensus, or the authors’ proposed practice framework.

## 2. Pathophysiology Relevant to Surgical Management

The tricuspid valve is most frequently and severely affected, developing fibrous retraction and immobility that produces severe regurgitation. The pulmonary valve is involved in up to 50% of cases, developing stenosis, regurgitation, or mixed disease [10]. Left-sided involvement is unusual because serotonin is effectively inactivated in the pulmonary vascular bed; exceptions include bronchial carcinoid and patent foramen ovale [11].

The fibrous plaques are histologically distinctive, comprising myofibroblasts, smooth muscle cells, and an extracellular matrix rich in collagen, deposited on the endocardial surface in a pattern that preferentially affects the downstream aspect of valve leaflets. Serotonin acts primarily through 5-HT2B receptors on valvular interstitial cells, stimulating mitogenesis and extracellular matrix production. This receptor-mediated mechanism explains the clinical observation that urinary 5-hydroxyindoleacetic acid levels correlate with the risk of developing CaHD, and underpins the rationale for somatostatin analogue therapy in slowing cardiac disease progression. The selective right-sided involvement is explained by the anatomical pathway: serotonin and tachykinins from hepatic metastases enter the inferior vena cava and reach right-sided structures in high concentration, while pulmonary vascular endothelium efficiently metabolises serotonin via monoamine oxidase, protecting left-sided structures. Left-sided carcinoid heart disease, while uncommon, should be actively sought in any patient with carcinoid syndrome and unexplained left-sided valvular pathology.

**Figure 1:**
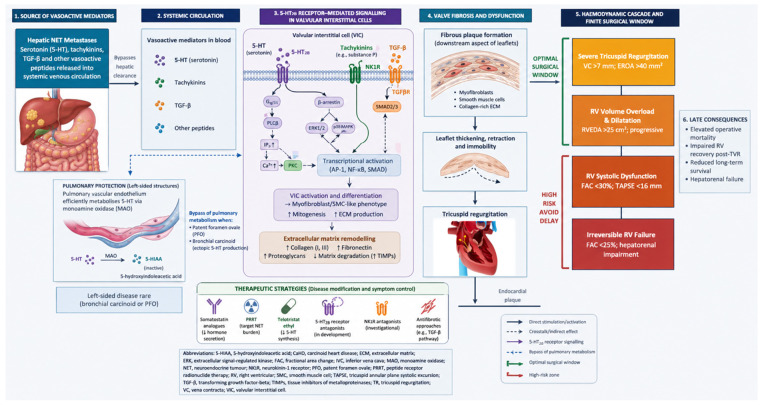
Pathophysiology of carcinoid heart disease. Vasoactive mediators released by hepatic neuroendocrine tumour metastases reach right-sided cardiac structures, inducing fibrous plaque deposition and progressive valve dysfunction. The optimal surgical window—before irreversible right ventricular dysfunction—is indicated. Serotonin (5-HT) acts via 5-HT2B receptors on valvular interstitial cells, stimulating mitogenesis and extracellular matrix (ECM) production through transforming growth factor-beta (TGF-β) signalling. Tachykinins and other vasoactive peptides contribute to the fibrotic process. The resulting fibrous plaques comprise myofibroblasts, smooth muscle cells, and collagen-rich ECM deposited preferentially on the downstream aspect of valve leaflets, causing retraction, immobility, and progressive regurgitation. Pulmonary vascular endothelium efficiently metabolises serotonin via monoamine oxidase (MAO), protecting left-sided structures except in the presence of patent foramen ovale (PFO) or bronchial carcinoid. The cascade from tricuspid regurgitation through right ventricular (RV) dilatation to irreversible RV failure and hepatorenal dysfunction defines the finite surgical window. 5-HIAA, 5-hydroxyindoleacetic acid; CaHD, carcinoid heart disease; FAC, fractional area change; IVC, inferior vena cava; NET, neuroendocrine tumour; PFO, patent foramen ovale; PRRT, peptide receptor radionuclide therapy; RV, right ventricular; TAPSE, tricuspid annular plane systolic excursion; TGF-β, transforming growth factor-beta; TR, tricuspid regurgitation.

The critical haemodynamic consequence is progressive RV volume and pressure overload. Sustained tricuspid regurgitation produces RV dilatation, secondary annular dilatation, hepatic congestion, and ultimately irreversible RV failure (Figure 1). This cascade defines the importance of surgical timing: once advanced RV dysfunction has occurred—characterised by severe dilatation, reduced fractional area change (FAC), and hepatorenal dysfunction—postoperative recovery is substantially impaired, even after technically successful valve replacement [7,12].

This pathophysiological cascade defines a finite surgical window during which intervention can be performed with acceptable risk. Identifying and acting within this window, before irreversible RV dysfunction and hepatorenal impairment have developed, requires structured echocardiographic surveillance and a proactive multidisciplinary approach rather than passive symptom-based referral.

## 3. Timing of Surgery

### 3.1. Inadequacy of Symptom-Based Referral

Multiple contemporary series suggest that symptom-based referral alone is associated with inferior outcomes [7,8]. Many patients with severe tricuspid regurgitation remain minimally symptomatic—due to compensatory RV dilatation—until ventricular dysfunction is advanced and surgical risk has risen substantially. The Mayo Clinic analysis of 195 patients over 40 years demonstrated perioperative mortality improving from approximately 10% overall to 6% in the modern era, with patients operated upon earlier showing substantially improved long-term survival (10-year actuarial survival 24%, limited predominantly by tumour progression) [5].

The era effect within this series is striking: perioperative mortality decreased from over 20% in the pre-2000 cohort to 6% in the modern era, reflecting improvements in surgical technique, critical care, earlier referral, structured perioperative octreotide protocols, and advances in oncological disease control. This temporal improvement provides the strongest observational evidence that a systematic approach to CaHD management substantially improves outcomes.

### 3.2. Right Ventricular Function as the Dominant Predictor

Preoperative RV function has emerged as the strongest reported predictor of operative and long-term survival in contemporary retrospective series. Nguyen and colleagues reported 240 patients (2000–2017) with 5% operative mortality in the modern era; preoperative RV dysfunction was independently associated with mortality on multivariate analysis [12]. Sawma and colleagues reported eight highly selected patients with early-stage disease (2010–2023) achieving 0% operative mortality and 86% 10-year survival, reinforcing the principle that timely intervention produces markedly superior results [13]. NT-proBNP elevation is independently associated with CaHD severity and is a useful serial biomarker alongside echocardiographic parameters to guide referral timing [14].

Beyond conventional echocardiographic parameters, newer imaging techniques offer incremental value in assessing RV function in CaHD. Right ventricular free-wall longitudinal strain assessed by speckle-tracking echocardiography has been shown to be reduced in patients with intestinal carcinoid disease independently of overt valvular involvement, suggesting subclinical myocardial impairment that precedes haemodynamically significant valve disease [15]. Furthermore, two-dimensional speckle-tracking echocardiography-derived myocardial strain, combined with the biomarker activin A, has been demonstrated to predict mortality in patients with carcinoid intestinal disease, providing prognostic information beyond that available from standard echocardiographic assessment [16]. Cardiac magnetic resonance imaging (CMR) provides complementary assessment, enabling accurate quantification of RV volumes and ejection fraction, characterisation of valve morphology including pulmonary valve disease that may be underestimated by transthoracic echocardiography, and detection of myocardial metastases and endocardial fibrosis using late gadolinium enhancement sequences [17,18]. A multimodality imaging approach incorporating conventional echocardiography, strain assessment, and CMR where available is therefore recommended for the comprehensive preoperative evaluation of RV function in CaHD patients being considered for surgical intervention.

### 3.3. Echocardiographic Triggers for Referral

**Figure 2:**
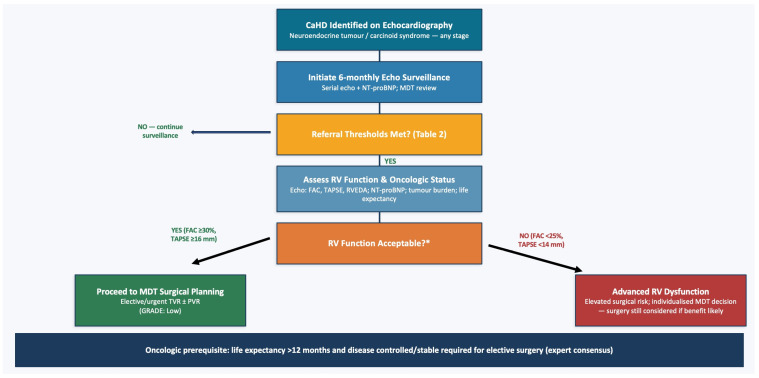
Proposed algorithm for the timing of cardiac surgery in carcinoid heart disease. * RV function thresholds—FAC: fractional area change; TAPSE: tricuspid annular plane systolic excursion; RVEDA: RV end-diastolic area. TVR = tricuspid valve replacement; PVR = pulmonary valve replacement; MDT = multidisciplinary team; NT-proBNP = N-terminal pro-B-type natriuretic peptide. GRADE evidence quality for referral thresholds: Low to Very Low. Thresholds represent expert consensus, not prospectively validated CaHD-specific cutoffs. The algorithm integrates echocardiographic parameters, right ventricular function assessment, NT-proBNP trajectory, and oncological disease status. Entry point: all patients with carcinoid syndrome and elevated urinary 5-HIAA should undergo baseline echocardiographic screening. Decision nodes are based on TR severity (severe: VC > 7 mm or EROA > 40 mm^2^), RV systolic function (FAC ≥ 30% representing the optimal surgical window; FAC < 25% indicating elevated operative risk), TAPSE (≥16 mm acceptable; <14 mm advanced dysfunction), rising NT-proBNP trajectory, and oncological disease status (controlled or stable with life expectancy > 12 months). The algorithm distinguishes between surveillance intervals (annual for normal baseline; 6-monthly for established valve thickening or mild regurgitation) and triggers for surgical referral. These thresholds are adapted from general valve disease guidelines and expert consensus and have not been prospectively validated in CaHD-specific cohorts. Referral threshold criteria are detailed in Table 2. 5-HIAA, 5-hydroxyindoleacetic acid; CaHD, carcinoid heart disease; EROA, effective regurgitant orifice area; FAC, fractional area change; NT-proBNP, N-terminal pro-B-type natriuretic peptide; RV, right ventricular; TAPSE, tricuspid annular plane systolic excursion; TR, tricuspid regurgitation; VC, vena contracta.

Current practice increasingly advocates referral based on imaging progression rather than symptoms alone (Table 1, Figure 2). Criteria integrating echocardiographic parameters (vena contracta > 7 mm, effective regurgitant orifice area > 40 mm^2^, FAC ≥ 30%, TAPSE ≥ 16 mm), NT-proBNP trajectory, and oncological status represent contemporary guidance for optimal surgical referral [7,19,20]. These thresholds are adapted from general tricuspid regurgitation and RV dysfunction criteria; they have not been prospectively validated in CaHD-specific cohorts and should be interpreted as consensus-informed guidance rather than evidence-based cutoffs.

The practical implication of these thresholds is that all patients with carcinoid syndrome and elevated urinary 5-HIAA should undergo baseline echocardiographic screening at diagnosis, with repeat imaging at 6–12-month intervals depending on the trajectory of echocardiographic findings and biomarker levels. Patients with normal baseline echocardiography and well-controlled tumour biology (stable or falling 5-HIAA, stable chromogranin A) may be rescreened annually. Those with established valve thickening or mild regurgitation require closer surveillance at six-monthly intervals, with referral for surgical assessment triggered by the imaging and biomarker criteria described above. This structured surveillance approach, while resource-intensive, is the only means of identifying the optimal surgical window before it has passed.

## 4. Operative Strategy

### 4.1. Tricuspid Valve Replacement

Tricuspid valve repair has yielded poor results in CaHD because the dominant pathology is leaflet fibrosis, retraction, and immobility rather than isolated annular dilatation; annuloplasty techniques are associated with early recurrence in published series [22]. Tricuspid valve replacement (TVR) is therefore required in the vast majority of patients. In the series by Connolly and colleagues, TVR was performed in all 195 patients; in the Nguyen series, TVR constituted the principal procedure in 240 cases [5,12].

Operative technique requires attention to the right coronary artery and the atrioventricular node conduction pathway. Suture placement with appropriate pledget buttressing of the fibrotic annulus is important, as degenerated tissue may hold sutures poorly. Permanent pacemaker implantation rates are higher in CaHD than in non-carcinoid tricuspid surgery, reflecting the fragility of the conduction tissue in the fibrotic annular environment. The choice of prosthesis size requires careful consideration: a prosthesis that is too small risks patient–prosthesis mismatch, which is poorly tolerated by the dysfunctional right ventricle, while oversizing risks conduction injury and paravalvular leak. Intraoperative transoesophageal echocardiography is essential to confirm prosthesis function before chest closure.

### 4.2. Pulmonary Valve Intervention

Pulmonary valve disease is frequently underestimated, partly because transthoracic echocardiographic assessment of the pulmonary valve is technically challenging and may underrepresent the severity of disease. Concurrent pulmonary valve dysfunction is present in up to 50% of patients at surgery, and systematic intraoperative inspection is therefore recommended [10]. Persistent uncorrected pulmonary valve disease after isolated TVR may impair RV recovery. Multiple centres advocate routine intraoperative inspection and a low threshold for concomitant pulmonary valve replacement (PVR); in the Nguyen series, omission of PVR in the presence of significant disease was associated with inferior RV functional recovery [12].

The rationale for a low threshold is that the haemodynamic cost of adding pulmonary valve replacement to an already-planned tricuspid procedure is modest—typically adding 20 min of cross-clamp time—whereas leaving significant pulmonary valve disease uncorrected imposes a persistent afterload and volume burden on an already-compromised right ventricle. Furthermore, reoperation for isolated pulmonary valve disease after prior tricuspid replacement carries substantially higher risk than concomitant correction at the index procedure.

### 4.3. Prosthesis Selection

Bioprosthetic valves are generally favoured in contemporary practice on the basis of anticoagulation avoidance (problematic given frequent oncological procedures and hepatic dysfunction), and the demonstrated feasibility of transcatheter valve-in-valve therapy for degenerated bioprostheses [23]. Veen and colleagues reported comparable early outcomes and three-year survival of 73% versus 56% for bioprosthetic and mechanical valves, respectively (non-significant) [23].

The durability of bioprosthetic valves in the tricuspid position is generally favourable, with freedom from structural valve degeneration exceeding 80% at 10 years in non-carcinoid populations. In CaHD patients, however, prosthetic valve degeneration may be accelerated by ongoing serotonin exposure if the underlying tumour remains active, though this concern is increasingly mitigated by effective somatostatin analogue therapy and PRRT. The emerging availability of transcatheter valve-in-valve therapy provides an additional safety net: patients who develop bioprosthetic degeneration can be treated percutaneously without the need for redo sternotomy, which is particularly valuable in this oncologically compromised population. This consideration substantially reinforces the preference for bioprosthetic over mechanical prostheses at the index operation.

### 4.4. Transcatheter Options

With increasing numbers of CaHD patients surviving long enough to develop bioprosthetic valve degeneration, transcatheter valve-in-valve therapy has emerged as an important option [24,25]. Khan and colleagues reported technically successful transcatheter pulmonary and tricuspid valve-in-valve replacement; Barbier and colleagues described a small case series with acceptable short-term outcomes, though follow-up data remain limited [25,26]. These approaches are particularly attractive in patients with high reoperative risk. Primary transcatheter TVR using dedicated systems is under clinical evaluation; current evidence in CaHD is absent, and surgical replacement remains the standard of care for operable patients. More recently, Barbier and colleagues reported the first dedicated series of transcatheter valve replacements in CaHD, comprising 15 procedures in nine patients (2021–2025) involving pulmonary, tricuspid, and aortic positions using SAPIEN 3, TOPAZ, and LUX devices, with 100% procedural success and significant improvement in NYHA class [26]. These emerging data, together with a recent comprehensive review of evolving transcatheter strategies in carcinoid syndrome [27], suggest that the therapeutic landscape is evolving rapidly, though longer follow-up and larger cohorts are required before transcatheter approaches can be recommended as alternatives to surgery in operable patients.

The anatomical and haemodynamic features of CaHD require cautious interpretation of transcatheter valve-in-valve outcomes. Carcinoid fibrous plaque deposition may distort the geometry of the previously implanted bioprosthesis, alter the landing zone for transcatheter devices, and create unpredictable interactions between the transcatheter valve frame and the rigid carcinoid-affected tissue. Furthermore, many CaHD patients have concurrent hepatic dysfunction that alters the pharmacokinetics of antiplatelet and anticoagulant agents required after transcatheter valve implantation. Despite these caveats, the accumulating case-based evidence supports valve-in-valve therapy as a valuable option for patients with degenerated bioprostheses who are at prohibitive surgical risk for redo sternotomy, and the availability of this option strengthens the argument for bioprosthetic valve selection at the index procedure.

## 5. Perioperative Management

The principal perioperative risk is carcinoid crisis: a life-threatening syndrome characterised by profound hypotension, flushing, bronchospasm, and dysrhythmia triggered by massive vasoactive mediator release during anaesthesia or surgery [28]. Continuous perioperative octreotide infusion (100–500 micrograms/hour, commenced 12–24 h preoperatively, maintained intraoperatively, continued 24–48 h postoperatively) is widely adopted as standard practice at experienced centres (Table 2) [29].

**Table 2:** Recommended perioperative management protocol.

Phase	Management	Source
Preoperative (12–24 h)	IV octreotide 100–500 mcg/h; depot injection as scheduled; avoid histamine-releasing agents	Baron 2020; Steeds 2019 [21,28]
Intraoperative	Maintain IV octreotide; vasopressin/phenylephrine for instability (avoid catecholamines); cell salvage if appropriate	Baron 2020; Steeds 2019 [21,28]
Postoperative (24–48 h)	Continue IV octreotide 24–48 h; resume depot analogue promptly; cardiac monitoring for arrhythmia and RV recovery	Baron 2020; Das 2023 [20,28]

IV, intravenous; RV, right ventricular.

Agents known to provoke histamine release—including morphine, atracurium, and succinylcholine—should be avoided. Haemodynamic instability should be treated with vasopressin or phenylephrine as first-line agents. Catecholamines should be minimised and used with caution, as they may paradoxically worsen vasodilation by stimulating tumour mediator release. Patients on long-acting somatostatin analogues should receive their usual depot injection as scheduled, with supplemental intravenous octreotide for the perioperative period [29].

Cardiopulmonary bypass management in CaHD requires specific attention. The initiation of bypass and the manipulation of the heart and great vessels during cannulation are recognised triggers for mediator release. Some centres supplement the bypass circuit prime with octreotide to maintain serotonin receptor blockade throughout perfusion. Weaning from bypass may be complicated by right ventricular dysfunction, particularly in patients with preoperatively impaired function; inotropic support with milrinone (which provides both inotropy and pulmonary vasodilation) and inhaled nitric oxide or prostacyclin for pulmonary vasodilation should be available. Noradrenaline should be used with caution, as catecholamine-mediated stimulation of tumour mediator release may worsen haemodynamic instability. Temporary right ventricular mechanical support has been reported in refractory cases but remains anecdotal.

## 6. Surgical Outcomes

Contemporary series consistently report that operative mortality has decreased substantially in experienced centres (Table 3). Connolly and colleagues reported 10% overall and 6% post-2000 mortality in 195 patients, with 10-year survival of 24% [5]. Mokhles and colleagues reported comparable outcomes in 19 patients, with 1-year survival of 71% and 5-year survival of 43%, with late mortality driven predominantly by NET progression [30]. Nguyen and colleagues reported 5% modern-era mortality in 240 patients [13]. Late mortality was predominantly related to NET progression rather than cardiac failure in patients undergoing timely intervention, though these observations derive from retrospective series at high-volume referral centres and may not be generalisable. Functional outcomes are favourable, with significant improvement in NYHA class, reduced hepatic congestion, and regression of RV dilatation on postoperative echocardiography.

The pattern of late mortality is informative: in patients undergoing timely surgery with preserved RV function, the dominant mode of death is tumour progression rather than cardiac failure, confirming that successful valve surgery effectively removes cardiac disease as a competing cause of mortality. This observation has important implications for patient counselling: operative intervention does not cure the underlying malignancy, but it does restore cardiac reserve, alleviate right-heart failure symptoms, and permit patients to tolerate subsequent oncological therapies; including hepatic-directed therapies, PRRT, and systemic chemotherapy, that would otherwise be contraindicated in the setting of decompensated right-heart failure.

## 7. Multidisciplinary Management

Optimal care requires the integration of cardiology, cardiac surgery, oncology, endocrinology, and specialist anaesthesia [26]. On current evidence, cardiac surgery is best considered when RV function remains preserved and oncological disease is controlled or stable, rather than waiting for further tumour response at the cost of progressive RV dysfunction [7,21]. Somatostatin analogue therapy should be reinitiated promptly postoperatively; PRRT can typically resume several weeks after uncomplicated cardiac surgery.

The sequencing of cardiac surgery relative to oncological therapy requires careful planning. In patients with progressive hepatic metastatic disease, hepatic-directed therapy (transarterial embolisation, radiofrequency ablation, or surgical debulking) may be considered before or after cardiac surgery depending on the relative urgency of each. In general, cardiac surgery takes priority when RV function is declining, because progressive right-heart failure limits the ability of the patient to tolerate the haemodynamic stress of hepatic interventions and systemic oncological therapy. Conversely, in patients with stable cardiac disease but rapidly progressive tumour burden, oncological treatment may appropriately precede cardiac intervention provided RV function is monitored closely during the interval. These decisions require case-by-case adjudication by the MDT and cannot be reduced to a simple algorithmic sequence.

## 8. Limitations

The evidence base comprises exclusively retrospective, single-centre series subject to referral and survivor bias. Referral bias is pervasive: patients reaching surgery were those who survived long enough and remained fit for operative assessment. The echocardiographic referral thresholds proposed derive from expert consensus rather than CaHD-specific prospective validation, as discussed further below. Formal meta-analysis is precluded by substantial heterogeneity in patient selection, era effects, and outcome reporting.

Several additional limitations warrant acknowledgement. Publication bias may affect the literature: favourable outcomes from experienced centres are more likely to be published than adverse outcomes. The echocardiographic thresholds for referral proposed in this review derive from expert consensus and extrapolation from general valve disease guidelines rather than prospective validation in CaHD-specific cohorts; they should be regarded as guidance rather than validated evidence-based cutoffs. This review was conducted by a single author without independent dual screening; while pre-specified inclusion criteria were applied, the risk of selection bias is greater than in dual-screened reviews. Finally, the clinical heterogeneity across published series—reflecting differences in centre volume, era, oncological treatment intensity, and patient selection—means that pooling of data across studies, even narratively, requires caution. Prospective multicentre registries with standardised data collection represent the most important future contribution to this field.

**Figure 3:**
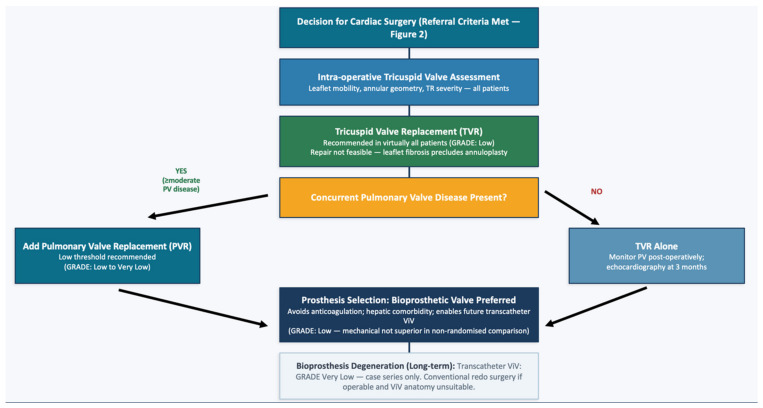
Proposed operative strategy algorithm for carcinoid heart disease. Decision points for tricuspid and pulmonary valve intervention, prosthesis selection, and the role of transcatheter valve-in-valve therapy for degenerated bioprostheses are illustrated. The algorithm commences with the decision for tricuspid valve replacement (TVR), which is required in the vast majority of CaHD patients given that repair yields poor results due to leaflet fibrosis and retraction. Intraoperative transoesophageal echocardiography (TOE) is essential to confirm prosthesis function. A low threshold for concomitant pulmonary valve replacement (PVR) is advocated where concurrent pulmonary valve disease is present, as leaving significant disease uncorrected imposes persistent afterload on the compromised RV. Bioprosthetic valves are preferred on the basis of anticoagulation avoidance and the feasibility of subsequent transcatheter valve-in-valve therapy. For patients developing bioprosthetic structural valve degeneration, transcatheter valve-in-valve replacement provides a less invasive alternative to redo sternotomy, particularly in those with prohibitive surgical risk. Primary transcatheter TVR using dedicated systems remains under clinical evaluation and is not yet standard of care. Referral threshold criteria are detailed in Figure 2. CaHD, carcinoid heart disease; PVR, pulmonary valve replacement; RV, right ventricular; TOE, transoesophageal echocardiography; TVR, tricuspid valve replacement.

Importantly, all proposed echocardiographic thresholds and algorithmic frameworks in this review (Figure 2 and Figure 3) lack prospective CaHD-specific validation and should be regarded as the authors’ proposed consensus-informed framework rather than evidence-based guidelines. The referral criteria are largely adapted from general tricuspid regurgitation and right ventricular dysfunction literature rather than derived from CaHD-specific prospective cohort studies, and this limitation must be acknowledged when applying these thresholds in clinical practice.

## 9. Future Perspectives

Several areas merit future investigation. First, prospective multicentre registries with standardised echocardiographic, biomarker, and outcome endpoints are essential to validate the proposed referral thresholds and algorithmic frameworks. Second, the role of RV free-wall longitudinal strain by speckle-tracking echocardiography deserves prospective evaluation as a tool for refining surgical timing; the demonstration that RV strain is reduced independently of overt valvular disease in carcinoid patients suggests potential for earlier identification of myocardial impairment before conventional parameters deteriorate [15,16]. Third, cardiac MRI may become increasingly important for comprehensive preoperative assessment, particularly for quantifying RV volumes and detecting pulmonary valve disease underestimated by echocardiography [17,18]. Fourth, the evolving landscape of transcatheter valve replacement using dedicated tricuspid and pulmonary valve devices offers the prospect of less invasive primary intervention in high-risk patients, complementing the established valve-in-valve approach for degenerated bioprostheses [26,27]. Finally, novel biomarkers such as activin A, which has been shown to predict mortality in carcinoid intestinal disease independently of established cardiac parameters, may complement NT-proBNP in the risk stratification algorithm and deserve validation in larger cohorts [16].

## 10. Conclusions

Retrospective evidence supports that CaHD is surgically treatable with clinically important symptomatic and survival benefit when intervention is appropriately timed. Operative timing, guided by echocardiographic and biomarker surveillance rather than symptoms alone, appears to be the most important modifiable determinant of outcome on current evidence. Comprehensive right-sided valve surgery—TVR with concomitant PVR where indicated—using bioprosthetic valves, combined with structured perioperative octreotide protocols, represents current best practice at experienced centres. Transcatheter valve-in-valve therapy offers a valuable option for degenerated bioprostheses. Prospective multicentre registries are needed to strengthen the evidence base for this rare condition.

The algorithms proposed in this review (Figure 2 and Figure 3) are intended as practical decision-support tools rather than prescriptive guidelines, and should be adapted to individual patient circumstances and institutional expertise. Their core principles—proactive echocardiographic surveillance, timely referral before irreversible RV dysfunction, comprehensive right-sided valve surgery, and structured perioperative endocrine management—represent the strongest consensus that the current evidence permits.

## Figures and Tables

**Table 1:** Echocardiographic and biomarker criteria guiding referral for surgical assessment.

Parameter	Threshold	Source
TR severity	Severe: VC > 7 mm or EROA > 40 mm^2^	Davar 2017; Otto 2021 [7,19]
RV end-diastolic area	Progressive dilatation: RVEDA > 25 cm^2^	Connolly 2015; Nguyen 2019 [5,12]
RV systolic function (FAC)	FAC ≥ 30% (optimal window); FAC < 25% (elevated risk)	Nguyen 2019; Møller 2003 [11,12]
TAPSE	≥16 mm (acceptable); <14 mm (advanced dysfunction)	Steeds 2019; Davar 2017 [7,21]
NT-proBNP	Elevated or rising on serial assessment	Bhattacharyya 2008 [14]
Oncological status	Controlled/stable disease; life expectancy > 12 months	Steeds 2019; Davar 2017 [7,21]

EROA, effective regurgitant orifice area; FAC, fractional area change; NT-proBNP, N-terminal pro-B-type natriuretic peptide; RV, right ventricular; RVEDA, right ventricular end-diastolic area; TAPSE, tricuspid annular plane systolic excursion; TR, tricuspid regurgitation; VC, vena contracta. Note: All thresholds are derived from retrospective cohort data and expert consensus, with supporting evidence extrapolated from general valve disease guidelines (Otto 2021 [19]). No CaHD-specific prospective validation exists for any threshold. Level of evidence: TR severity and RVEDA thresholds—retrospective cohort data (Connolly 2015; Nguyen 2019 [5,12]); FAC and TAPSE thresholds—expert consensus extrapolated from general RV dysfunction criteria (Davar 2017; Steeds 2019 [7,21]); NT-proBNP—biomarker validation study in CaHD (Bhattacharyya 2008 [14]); oncological status—expert consensus (Steeds 2019; Davar 2017 [7,21]).

**Table 3:** Contemporary surgical outcomes in carcinoid heart disease.

Study	*n*	Procedure	Mortality	Survival	Key Finding
Connolly 2015 [5]	195	TVR ± PVR	10% (6% post-2000)	1-yr 69%; 5-yr 35%; 10-yr 24%	Era effect; early referral improves survival
Nguyen 2019 [12]	240	TVR ± PVR	5% (modern era)	1-yr 69%; 5-yr 34%	RV dysfunction strongest mortality predictor
Sawma 2025 [13]	8	TVR + PVR	0%	5-yr/10-yr 86%	Selected early-stage; confirms early-referral benefit
Mokhles 2012 [30]	19	TVR ± PVR	10%	1-yr 71%; 5-yr 43%	Late mortality driven by NET progression
Veen 2020 [23]	49	TVR (bio vs. mech)	NR by type	3-yr: 73% vs. 56% (ns)	Bioprosthetic comparable; no mechanical advantage

bio, bioprosthetic; mech, mechanical; NET, neuroendocrine tumour; NR, not reported; ns, non-significant; PVR, pulmonary valve replacement; RV, right ventricular; TVR, tricuspid valve replacement.

## Data Availability

No new data were created or analysed in this study. Data sharing is not applicable to this article.
